# Observation as a treatment strategy for advanced renal cell carcinoma—a call for prospective validation

**DOI:** 10.3389/fonc.2012.00155

**Published:** 2012-10-31

**Authors:** Rosalie Fisher, Alexandra Pender, Kiruthikah Thillai, Simon Chowdhury, Lisa Pickering, Komel Khabra, Martin Gore, James Larkin

**Affiliations:** ^1^Clinical Research Fellow, Department of Medical Oncology, The Royal Marsden HospitalLondon, UK; ^2^Specialist Registrar, Department of Medical Oncology, The Royal Marsden HospitalLondon, UK; ^3^Clinical Research Fellow, Department of Medical Oncology, Guy's and St. Thomas' HospitalLondon, UK; ^4^Consultant Medical Oncologist, Department of Medical Oncology, Guy's and St. Thomas' HospitalLondon, UK; ^5^Consultant Medical Oncologist, Department of Medical Oncology, The Royal Marsden HospitalLondon, UK; ^6^Statistician, The Royal Marsden HospitalLondon, UK; ^7^Professor of Medical Oncology, Department of Medical Oncology, The Royal Marsden HospitalLondon, UK

A number of cancers can follow an indolent clinical course, even when the disease is at an advanced stage. For example, this pattern can be observed in some patients with renal cell carcinoma (RCC), breast cancer, and low grade non-Hodgkin lymphoma (NHL). When systemic treatments for these conditions are palliative, chronic and often toxic, there is an argument for deferring therapy until there is a clinically relevant burden of disease, at which time the side effects of treatment are counter-balanced by relief of symptoms and disease control. A randomized trial of “watchful waiting” compared to immediate chemotherapy treatment in asymptomatic patients with low-grade NHL found that overall survival between these two groups was the same, and the authors proposed that this approach might be particularly useful in elderly patients (Ardeshna et al., 2003). Furthermore, preliminary results of a randomized trial of immediate rituximab (an anti-CD20 monoclonal antibody) versus a watch and wait strategy in patients with asymptomatic follicular lymphoma were presented and indicate that rituximab significantly delays the time to initiation of new therapy such as chemotherapy or radiotherapy (Ardeshna et al., 2010). It is important to note that rituximab has a favorable side effect profile, and the most powerful argument for a watchful waiting approach is freedom from debilitating side effects and preservation of quality of life for patients.

Prospective evidence for an initial observational strategy in other solid tumor types is limited, even though it is common in clinical practice. It is well recognized that a subgroup of patients with advanced RCC has slowly progressive metastatic disease over a number of years. Metastatic RCC (mRCC) was considered refractory to systemic therapy for many years, but there are now seven so called “targeted” agents approved for this condition, which target the critical vascular endothelial growth factor (VEGF) and mammalian target of rapamycin (mTOR) pathways, leading to inhibition of angiogenesis and cell survival and proliferation. All seven drugs have been shown in randomized clinical trials to significantly improve clinical outcomes for patients with mRCC, but they are non-curative and associated in general with moderate toxicity. Up to 20% of patients appear to be primarily refractory to these treatments (Rini and Flaherty, 2008), and almost all patients will eventually become resistant to an individual drug, necessitating sequential, chronic therapy. Because of the potential for substantial toxicity, a key question in this field is the optimal time to start treatment. It has been inferred from a number of sources, including a randomized discontinuation trial of sorafenib (Ratain et al., 2006), that treatment delays do not have an adverse impact but there are no published data to support this contention.

Recently, we conducted a retrospective cohort study of patients treated at two centers to evaluate the clinical outcomes of those patients with metastatic renal cell cancer treated in the “targeted therapy era,” in who first line systemic therapy was deliberately deferred. Sixty-two patients with mRCC who had a planned period of observation prior to starting first line therapy, because of asymptomatic or slowly progressive disease, were included and the primary objective was to determine the progression free survival (PFS) of patients on deferred first line systemic therapy.

All but one patient had favorable or intermediate risk disease (63% and 36% respectively), as defined by Heng et al. (2009). On average, patients with mRCC were observed for 18.7 months (95% CI 14.5–22.0 months). After a period of observation, 39 patients were treated with sunitinib, 18 with interferon, and 5 with other agents such as mTOR inhibitors. Overall, the median PFS for patients on first line therapy was 9 months (95% CI 8.1–10.1 months). Patients treated with sunitinib after observation also had a median PFS of 9 months (95% CI 8.1–9.9 months), and those treated with interferon had a median PFS of 6.7 months (95% CI 0.7–12.7 months). Median overall survival, defined as the time from starting first line treatment to death, was 25.2 months for all patients (95% CI 8.0–42.4 months), 17.4 months (95% CI 11.6–23.2 months) in the sunitinib group, and 37.6 months (95% CI 2.6–72.5 months) in the interferon group.

Thus, in this cohort of patients with indolent, favorable or intermediate prognosis mRCC, first line systemic therapy was deferred by an average of more than 18 months and median PFS and overall survival times were comparable to those observed in the pivotal phase III and expanded access trials of sunitinib (Motzer et al., 2007; Gore et al., 2009).

Retrospective data such as these are limited and clearly reflect selection bias. However, they suggest that this practice in mRCC is reasonable and does not compromise outcome, and in our view, there are compelling reasons for observational strategies to be prospectively, rigorously studied in this and other tumor types. This would provide an opportunity to evaluate longitudional quality of life data using tools such as Quality-adjusted Time Without Symptoms or Toxicity (Q-TWiST), which incorporates duration of survival and quality of life experienced into a single endpoint (Cole et al., 2004). It is possible that surveillance only for advanced cancer results in increased patient anxiety, and thus harms quality of life, but this should be prospectively assessed. Routine collection of tumor tissue from these patients would enable investigation and validation of biomarkers predictive of an indolent clinical course, and importantly, this information could be extrapolated for use in the non-metastatic disease setting. For example, observation may also be appropriate in those patients with incidental small renal masses, particularly in the presence of co-morbidities. Finally, an observational strategy might result in more efficient use of limited financial resources, a problem which is now faced by almost all developed countries.
